# Production and bioprocessing of camptothecin from *Aspergillus terreus*, an endophyte of *Cestrum parqui*, restoring their biosynthetic potency by *Citrus limonum* peel extracts

**DOI:** 10.1186/s12934-022-02012-y

**Published:** 2023-01-06

**Authors:** Ashraf S. A. El-Sayed, Nelly M. George, Amira Abou-Elnour, Rasha M. El-Mekkawy, Marwa M. El-Demerdash

**Affiliations:** Enzymology and Fungal Biotechnology Lab, Botany and Microbiology Department, Faculty of Science, Zagazig University, Zagazig, Egypt

**Keywords:** Camptothecin, Anticancer activity, *Aspergillus terreus*, Biosynthetic stability, LC/MS analyses

## Abstract

The metabolic potency of fungi as camptothecin producer elevates their prospective use as an industrial platform for commercial production, however, the loss of camptothecin productivity by fungi with the storage and subculturing are the major obstacle. Thus, screening for endophytic fungal isolates inhabiting ethnopharmacological plants with an obvious metabolic stability and sustainability for camptothecin biosynthesis could be one of the most feasible paradigms. *Aspergillus terreus* ON908494.1, an endophyte of *Cestrum parqui* was morphologically and molecularly verified, displaying the most potent camptothecin biosynthetic potency. The chemical identity of *A. terreus* camptothecin was confirmed from the HPLC, FTIR and LC–MS/MS analyses, gave the same molecular structure and mass fragmentation patterns of authentic one. The purified putative camptothecin displayed a strong anticancer activity towards HepG-2 and MCF-7 with IC_50_ values 0.96 and 1.4 µM, respectively, with no toxicity to OEC normal cells. As well as, the purified camptothecin displayed a significant antifungal activity towards fungal human pathogen *Candida albicans*, *Aspergillus flavus*, and *A. parasiticus,* ensuring the unique structural activity relationships of *A. terreus* camptothecin, as a powerful dually active anticancer and antimicrobial agent. The camptothecin productivity of *A. terreus* was maximized by bioprocessing with Plackett–Burman design, with an overall 1.5 folds increment (170.5 µg/L), comparing to control culture. So, the optimal medium components for maximum yield of camptothecin by *A. terreus* was acid why (2.0 mL/L), Diaion HP20 (2.0 g/L), Amberlite XAD (2.0 g/L), dextrin (5.0 g/L), glucose (10.0 g/L), salicylic acid (2.0 g/L), serine (4.0 g/L), cysteine (4.0 g/L) and glutamate (10.0 g/L), at pH 6 for 15 days incubation. By the 5th generation of *A. terreus*, the camptothecin yield was reduced by 60%, comparing to zero culture. Interestingly, the productivity of camptothecin by *A. terreus* has been completely restored and over increased (210 µg/L), comparing to the 3^rd^ generation *A. terreus* (90 µg/L) upon addition of methanolic extracts of *Citrus limonum* peels, revealing the presence of some chemical signals that triggers the camptothecin biosynthetic machinery. The feasibility of complete restoring of camptothecin biosynthetic-machinery of *A. terreus* for stable and sustainable production of camptothecin, pave the way for using this fungal isolate as new platform for scaling-up the camptothecin production.

**Supplementary Information:**

The online version contains supplementary material available at 10.1186/s12934-022-02012-y.

## Introduction

Camptothecin derivatives are the third common anticancer drugs after Taxol and Vinca monoterpene indole alkaloids “vincristine, vinblastine” [1], with a broad-range anticancer activity against various types of tumors cells namely liver carcinoma, tumors of head and neck [2], stomach and bladder cancer [3]. Camptothecin was firstly extracted from the bark of *Camptotheca acuminate* (Nyssaceae) in China [4]. The potential anticancer activity of camptothecin elaborates from its affinity to bind with topoisomerase I, an indispensable enzyme in DNA transposition, transcription, DNA repair processes and replication by relaxation of DNA supercoiling [4]. Camptothecin triggers the apoptosis in various cancer cells by interfering with topoisomerase I and DNA complex resulting in a stabilized ternary complex, preventing DNA re-ligation, causing DNA degradation, and ultimate cell death [5]. With the broad range applications of camptothecin, the major hurdles impedes the clinical applications of this compound are: (1) Low water solubility with severe gastrointestinal toxicities of the core camptothecin compound [6], however, a recent development in preparation of a new chemical derivatives of camptothecin “10-hydroxycamptothecin, Topotecan, Hycamtin, Irinotecan, and Camptosar” [1], with high water solubility as approved by FDA [7]; and (2) The miniscule yield of camptothecin core from its natural source “*C. acuminata*” with the heavy demand of this compound resulted in a destructive harvesting of this plant in China and India that has a negative effect on their natural ecosystem [8, 9]. Naturally, the plant-derived bioactive compounds are usually of low abundance, difficulty in extraction, steric complexity, bulky compounds and diverse aromaticity [8–10]. Thus, searching for alternative approach with higher camptothecin productivity is the current challenge.

Fungal endophytes are considered as an untapped reservoir of unique biologically active compounds [10–15]. The rationality of the prospective applications of endophytic fungi for production of bioactive secondary metabolites elaborates from their fast growth, accessibility of bulk biomass production, independence on the environmental conditions and feasibility of metabolic manipulation [10–13, 15–17]. The metabolic biosynthetic potency of endophytic fungi for camptothecin production raises the hope for commercial production of this lead compound [10, 12, 18, 19]. Camptothecin was firstly isolated from *Entrophospora infrequens* “an endophyte of *Nothapodytes foetida*” [20–22], followed by several reports ensuring the metabolic potency of various endophytic fungal isolates as camptothecin producers [8, 16, 23–28]. However, the low yield of fungal camptothecin, in addition to the loss of camptothecin productivity with the storage and repetitive subculturing are the main challenge that halts their further industrial application [8, 16, 23, 25, 27, 28]. Among the medicinal plants, *Cestrum* spp. have been reported to have a massive amounts of fragrant oils, volatile compounds, phenolic compounds an various bioactive molecules [29, 30]. However, there is reports describing the microbial and physiological identities of the endophytic fungi from the *Cestrum* spp. especially for production of bioactive compounds particularly camptothecin. Thus, screening for the endophytic fungal isolates inhabiting *Cestrum* spp., and assessing their metabolic potency for sustainable and stable biosynthetic machinery of camptothecin was the objective of this study.

## Materials and methods

### Collection of the plant samples and isolation of endophytic fungi

Fresh leaves and twigs of *Cestrum diurnum, C. elegans, C. nocturnum* and *C. parqui* were collected from Al Orman botanical garden, Giza province, Egypt, in April/ 2019, these plant materials washed thoroughly with running sterile. The plant materials were segmented into small parts, surface sterilized with 70% ethyl alcohol for 1 min, and 2.5% sodium hypochlorite for 2 min, placed on the surface of potato dextrose agar (PDA) supplemented with ampicillin (1 μg/mL), incubated for 7 days at 30 °C [15, 18]. The recovered fungal isolates were morphologically identified according to their macroscopical and microscopical features [31, 32]. The evolved fungal hyphal tips were collected and purified on PDA, and kept as slope cultures at 4 °C for further uses.

### Screening for the most putative recovered isolated fungi

The recovered fungal isolates were screened for camptothecin production by growing on potato dextrose broth (PDB) medium (Extract of 200 g potato and 20 g glucose per liter) (BD, Difco, Cat. # DF0549-17-9). Each fungal isolate (2 agar plugs of 5 mm) of 6 days old PDA culture were inoculated into 50 mL PDB medium/250 mL Erlenmeyer flask, incubated at 30 °C for 15 days. Three biological replicates of each fungal isolate were conducted. After incubation, the cultures were filtered by sterile cheesecloth, and the filtrates were centrifuged at 5000 rpm to remove any particulates, and used for camptothecin extraction with CHCl_3_: MeOH (4:1) [10, 16, 33]. The organic phase was concentrated by a rotary evaporator to give a crude oily extract. The extract was fractionated on TLC with Merck 1 mm (20 × 20 cm) pre-coated silica gel plates (TLC Silica gel 60 F254, Merck KGaA, Darmstadt, Germany) with developing solvent system; chloroform: methanol (9:1, v/v) [16, 21]. The plates were visualized by UV illumination at 254 nm, and the putative camptothecin spots gave the same blue color, and relative mobility of authentic one (Cat.#7689-0-3-4) were considered. The intensity of putative spots was determined by the Image J software package referencing to known concentrations of authentic sample.

The putative spots of silica containing camptothecin were scraped-off, dissolved in methanol for camptothecin extraction [10]. The purity and concentration of camptothecin were determined by HPLC (YOUNG In, Chromass, 9110+ Quaternary Pump, Korea) with reverse-phase C18 column (Eclipse Plus C18 4.6 mm × 150 mm, Cat. #959963-902), using isocratic mobile phase methanol/ water (60:40 v/v) at a flow rate 1.0 mL/min for 20 min, scanned by photodiode array detector (DAD). The identity and concentration of the putative sample of camptothecin were confirmed from retention time and peak area, normalizing to the authentic one at λ360 nm [34, 35].

### UV–Vis, FT-IR, and LC–MS analyses

The putative spots of camptothecin, were scraped from the silica plate, dissolved in methanol, and scanned by UV–Vis spectrophotometer (RIGOL, Ultra-3000 Series) at λ300–400 nm. The concentration of the putative camptothecin was determined comparing to authentic concentration of camptothecin, using methanol as blank baseline. The FT-IR spectra of camptothecin were analyzed with a Bruker FT-IR Spectrometer in a range of 400–4000 cm^−1^ with KBr pellets. The chemical identity of camptothecin was verified by liquid chromatography tandem mass spectrometry (LC–MS/MS) with Thermo Scientific LCQ Deca mass spectrometer and Hypersil Gold C18 column, with an electrospray source in positive and negative ion modes. The mobile phases A (0.1% formic acid), and B (acetonitrile in 0.1% formic acid) were used [10, 34, 35]. The gradient elution system was 2–98% mobile phase B over 30 min at a flow rate of 0.2 mL/min for 40 min. The chemical identity of the resolved signals was determined regarding their retention times and mass spectral fragmentation pattern, regarding to the authentic camptothecin.

### Molecular identification of the recovered endophytic fungi

The identity of the potent camptothecin producing fungi was molecularly confirmed from the sequence of their internal transcribed spacers (ITS) [36]. The fungal genomic DNA (gDNA) was extracted by CTAB reagent and used as a template for PCR with the primer set; ITS5 5ʹ-TCCTCCGCTTATTGATATGC-3ʹ, ITS4 5ʹ-GAAGTAAAAGTCGTAACAAGG-3ʹ. The PCR reaction mixture consists of 10 µL of 2× PCR master mixture (i-Taq™, Cat. No. 25027), 1 µL of gDNA, primers (10 pmol), and completed to 20 µL, and the PCR was programed at initial denaturation 94 °C for 2 min, denaturation 94 °C for 30 s, annealing 55 °C for 20 s, extension 72 °C for 40 s, for 35 cycles, and 2 min final extension at 72 °C [37]. The PCR amplicons were sequenced by Applied Biosystems Sequencer, HiSQV Bases, and the sequence was non-redundantly BLAST searched on NCBI, imported into MEGA 7.0 software and aligned by Clustal W muscle algorithm [37] and the phylogenetic relatedness was created by the neighbor-joining method [38].

### Bioprocess optimization of camptothecin production by Plackett–Burman design

Various physicochemical parameters, acid whey, malt extract, potato starch, methyljasmonate, tryptamine, peptone, dextrin, tryptone, glucose, salicylic acid, tryptophan, serine, cysteine, pyruvate, phenylalanine, glutamate and fluconazole were optimized by Plackett–Burman design to maximize the yield of camptothecin from the target fungus [10, 34, 35]. The nineteen parameters were screened by two variables of Plackett–Burman design, each represented by high (+ 1) and low (− 1) levels, according to the first order reaction:

$$Y = \beta 0 +\sum \beta iXi$$

where, Y is the predicted yield of camptothecin, Xi is an independent variable, *b*i is the linear coefficient, and *b*0 is the model intercept. Triplicates for each run were conducted, and the average camptothecin yield was used as the main response.

### Metabolic biosynthetic stability of *A. terreus* camptothecin in response to subculturing, and the potency restoring its biosynthetic potency

The metabolic stability of *A. terreus* for production of camptothecin in response to successive subculturing has been estimated. The axenic fungal isolate “first isolate” has been sub-cultured as a slope culture on PDA for 10 days at 30 °C, followed by subsequent subculturing at the same conditions till the 8th generation. The camptothecin productivity by the different cultures was estimated at the same above conditions.

Induction of the biosynthetic machinery of fungal secondary metabolites in response to addition of some plant-derived natural compounds, and metabolic intermediates, could be crucial intermediates on biosynthesis of complex metabolites [10]. The effect of methanolic extract of fresh *C. limonum* peals (10 g/50 mL methanol) on modulating the CPT productivity by *A. terreus* was evaluated. Ten grams of fresh lemon peel were soaked in methanol overnight, the extracts were filtered, centrifuged at 5000 rpm, and concentrated by rotary evaporator. The methanolic extracts (1 mL) were added to the fungal cultures after 4 days of incubation, and the cultures were incubated for 15 days under the experimental conditions, camptothecin was extracted and quantified as described above.

### Antiproliferative activity of the purified camptothecin from *A. terreus*

The antiproliferative activity of extracted camptothecin from *A. terreus* was evaluated against the different cell lines; liver carcinoma (HepG-2) and breast carcinoma (MCF-7) was determined by 3-(4,5-dimethylthiazol-2-yl)-2,5-diphenyl tetrazolium bromide (MTT) assay [39]. The microtiter plate was seeded with 10^3^ cells per well, incubated for 12 h at 37 °C, each well was amended with different camptothecin concentrations, the plate was incubated for 48 h. The MTT reagent (25 µL) was added, and the developed purple color of formazan complex was measured at λ_570_ nm after 2 h. The IC_50_ value was expressed by the camptothecin concentrations suppressing the growth of 50% of the initial number of cells, normalizing to positive controls.

### Fungal deposition

The ITS sequence of the isolate *A. terreus* EFBL-CP, an endophytes of *C. parqui*, was deposited into the genbank with Accession No. ON908494.1.

## Results and discussion

### Isolation, and screening for camptothecin-producing endophytic fungi inhabiting different medicinal plants

**Fig. 1:**
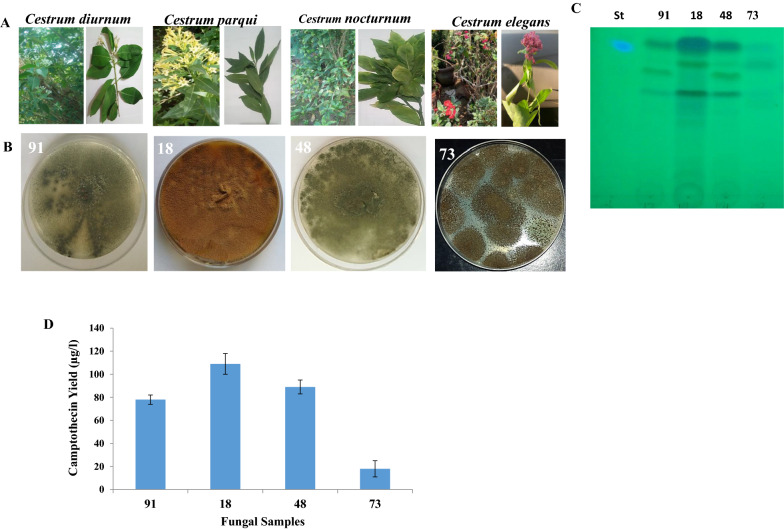
Camptothecin producing fungal endophytes from *Cestrum* species*; C. diurnum, C. elegans, C. nocturnum* and *C. parqui*. **A** Morphological view of leaves and twigs of *Cestrum* spp. **B** Potent Camptothecin producing endophytes; *Aspergillus niger* (73), *A. terreus* (18), *A. fumigatus* (48) and *A. flavus* (91). **C** Chromatographic analysis (TLC) of the extracted Camptothecin from the most potent fungal isolates comparing to authentic Camptothecin. **D** Yield of Camptothecin from the selected fugal isolates

Four medicinal plants; *Cestrum diurnum*, *C. elegans*, *C. nocturnum* and *C. parqui*, were selected as source for the experimental endophytic fungi. Thirty-one fungal isolates were isolated from the leaves and stems of the tested plants on PDA media. These fungal isolates were morphologically identified based on their microscopical features according to the universal keys, and the isolates were mainly belonging to the genus *Aspergillus*. The productivity of CPT by the recovered fungal isolates was assessed by growing on PDB, incubated at the standard conditions for 15 days at 30 °C, and then CPT was extracted, checked and quantified by TLC and HPLC. From the screening profile, *Aspergillus terreus* “an endophytes of the leaves of *Cestrum parqui*” had the highest CPT productivity (110 µg/L), followed by *A. terreus* “an endophyte of *C. nocturnum”* (96 µg/L) (Fig. 1) (Additional file 1: Table S1). As well as, several isolates of *A. flavus* inhabiting leaves and stems of *C. diurnum* and C. *elegans* exhibiting a mild camptothecin productivities (40–60 µg/L). Several paradigm of screening for camptothecin productivity has been reported by our previous studies [10, 34]. Interestingly, isolates of *Aspergillus terreus* as endophytes of from different plants have been reported frequently camptothecin producers [10], ensuring the efficiency of the molecular machinery of camptothecin productivity by *A. terreus*, as common mechanisms for plant defense, and common fungal-plant interaction. The difference on the camptothecin productivity of the same fungal species inhabiting different plant host ensuring the role of plant-fungal interaction, fungal-microbiome interactions, that may modulate the biosynthetic machinery, molecular expression of the genes encoding camptothecin by the specific fungal isolate. So, the each plant has not only own their endogenous microbiome but also each endophyte has a specific pattern of metabolic and physiological behavior.

### Molecular identification of the potent fungal isolate producing camptothecin

**Fig. 2:**
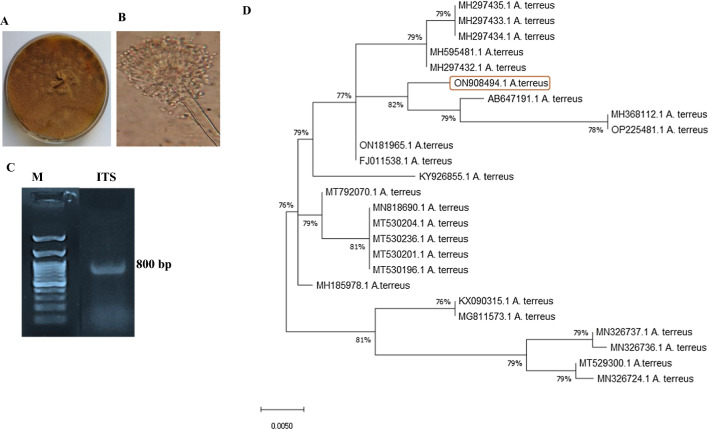
Morphological and molecular identification of most potent fungal endophyte from *C. parqui.* **A** Morphological features of *Aspergillus terreus* ON908494 grown on PDA. **B** Microscopical view of *A. terreus* at 1000× by Light Microscope. **C** PCR amplicon of the ITS region of the potent fungal isolate by 1% agarose gel electrophoresis with 1 kb DNA ladder (Cat. # PG010-55DI). **D** Molecular phylogenetic analysis of *A. terreus* ON908494 isolates by Maximum Likelihood method

The morphological identity of the potent camptothecin producing isolate “*Aspergillus terreus”* has been further identified based of on the sequence of their internal transcribed spacer (ITS) flanking the 5.8S region (ITS1-5.8S-ITS2 rDNA). The genomic DNA of the fungal isolate was extracted and used as PCR template, the size of PCR amplicon was about 800 bp (Fig. 2). The amplicon was purified, sequenced and non-redundantly BLAST searched on the NCBI nucleotide database, displaying a 99.9% similarity with *Aspergillus terreus* isolates with zero E-value and 98–100% query coverage. The sequence of the ITS region of *A. terreus* was deposited into the genbank under accession number ON908494.1. The sequence quality was visually inspected from the sequence chromatogram. For the multiple sequences alignment, FASTA sequences were imported into MEGA X software and aligned with Clustal W muscle algorithm. The phylogenetic tree of the target sequences was constructed with neighbor-joining method of MEGA X with 1000 bootstrap replication.

### Chromatographic and spectroscopic validation of chemical identity of camptothecin

**Fig. 3:**
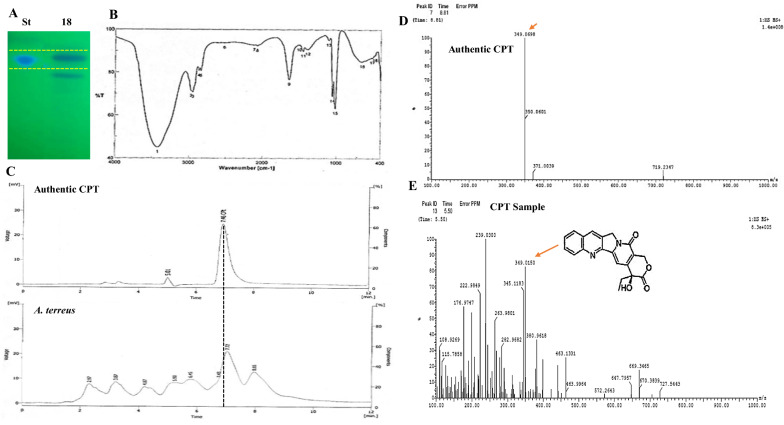
Chemical analysis and antiproliferative activity of extracted Camptothecin from *A. terreus*. **A** TLC chromatogram of putative camptothecin, the putative target spots were scraped-off from the TLC plates and used for further spectroscopic and chromatographic analyses. **B** The FT-IR spectral analysis of the putative camptothecin. **C** HPLC chromatogram of the purified sample comparing to the authentic one. LC–MS/MS analysis of the putative camptothecin (**D**, **E**) normalizing to the authentic one

The chemical identity of the putative CPT from *A. terreus* was confirmed from the HPLC, HNMR, CNMR, FT-IR and LC–MS analyses, comparing to the authentic sample. After cultural incubation, camptothecin was extracted, fractionated by TLC, and the putative silica gel spots of camptothecin gave the same mobility and color upon illumination at λ264 nm (Fig. 3A), were scraped-off and dissolved in methanol for further chemical analyses. The identity of putative sample was confirmed from HPLC analysis, from the HPLC chromatogram the putative sample gave the same retention time of the authentic camptothecin (7.42 min) (Fig. 3). As well as, the maximum absorption peak of the extracted camptothecin of *A. terreus* was reported at λ_290_ nm, that was identical to the absorption optima of authentic camptothecin. As well as, the chemical identity of *A. terreus* camptothecin was verified from the HNMR, displaying the same resolved signals of the authentic one, that were distributed between 1.0 and 8.0 ppm, with three proton signals resolved at 1.0–2.5 ppm corresponding to methyl, acetate and acetylene groups.

The chemical identity of the extracted camptothecin was confirmed from the FT-IR analysis, the sample displayed distinct peaks at 3406.6 and 3393.3 cm^−1^, assigned for the hydroxyl (OH) and amide group stretches, respectively. The apparent Peaks at 2923.56, 1729.83 and 1604.5 cm^−1^ were assigned to the aliphatic CH, ester groups and aromatic rings stretches. Also, the obvious stretching of the carboxyl functional group was peaked at 1268.9 cm^−1^, while peak at 1029.8 cm^−1^ was assigned for the aromatic C and H blends (Fig. 3B).

The chemical structure of the extracted camptothecin sample has been confirmed by LC–MS analysis positive mode. From the LC–MS/MS analysis, the sample gave the same molecular mass to charge ratio (349.02 m/z), in addition to the same molecular fragmentation pattern of authentic camptothecin (Fig. 3D, E). From the profile of the first mass spectra, a peak at retention time 5.5 min with a molecular ion peak at m/z 349 [M + H] + corresponding to the molecular formula C20H16N2O4 in addition to other diagnostic peaks of camptothecin alkaloid was resolved. Coincident patterns of chromatographic and spectral analyses for validating the chemical identity of camptothecin from *Camptotheca acuminata* [4], *A. terreus* “endophyte of *Ficus elastica* [10], *A. flavus* [34], *Penicillium chrysogenum* [35], were resolved. From the TLC, HPLC, FT-IR, LC–MS/MS, and UV-absorption spectra, the putative *A. terreus* sample has been chemically verified as camptothecin.

### The antiproliferative activity of *A. terreus* camptothecin

**Fig. 4:**
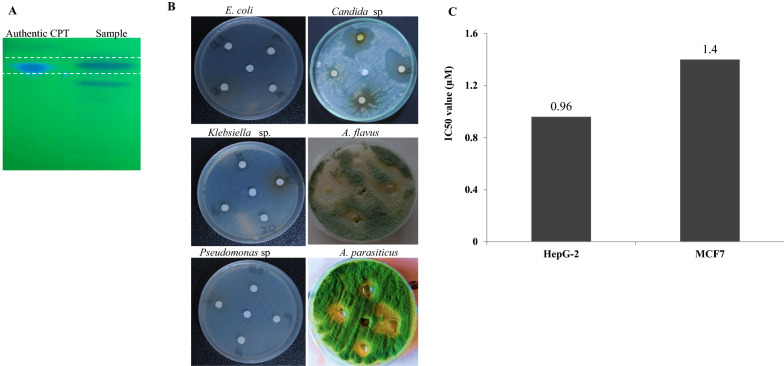
Antimicrobial activity of the extracted camptothecin from *A. terreus* towards various pathogenic bacteria and fungi. **A** The putative samples of camptothecin was extracted from the TLC. **B** Activity of extracted camptothecin against various bacteria “*E. coli*, *Klebsiella* sp. and *Pseudomonas* sp.”, and fungi “*Candida* sp., *Aspergillus flavus* and *A. parasiticus”.* **C** Antiproliferative activity of *A. terreus* camptothecin towards liver tumor cells (HepG-2) and breast adenocarcinoma (MCF7)

The antiproliferative activity of *A. terreus*-derived camptothecin was evaluated against various cell lines; liver tumor cells (HEPG2) and breast adenocarcinoma (MCF7). Different concentrations of purified camptothecin were amended to the medium, and the cell viability was measured. From the IC_50_ values, the purified *A. terreus* camptothecin had a significant activity towards HepG-2 (0.96 µM) and MCF (1.4 µM) (Fig. 4). The antiproliferative activity of *A. terreus* camptothecin was consistent with the camptothecin derived from various plants and fungal sources [3, 10, 16, 27, 34, 35, 40].

### Antimicrobial activity of *A. terreus* camptothecin

The antimicrobial activity of the bioactive secondary metabolites has been used as a preliminary assay for predicting their proliferative activity [41]. After incubation of the tested fungal cultures, camptothecin was extracted, fractionated by TLC, and the putative camptothecin-containing silica gel spots were scraped-off, and dissolved in methanol for biological activity analyses, against various microorganisms. From the antimicrobial activity as revealed from the diameter of the inhibition zones (Fig. 4), the putative camptothecin displayed a powerful antifungal activity towards *Candida* sp. (1.5 mm), *A. parasiticus* (2.6 mm) and *A. flavus* (0.6 mm)*,* unlike to its mild antibacterial activity. Interestingly, the higher antifungal activity of the extracted putative *A. terreus* camptothecin was synchronized with their antiproliferative activity towards the tested cell lines, ensuring the structural activity relationships of putative camptothecin samples towards the Topoisomerase I from human and fungal cells [24, 41, 42]. The higher antifungal and anticancer activities of camptothecin might be due to the proximity of amino acid sequence and tertiary structures of topoisomerase I from mammalian cells and fungal cells, than bacterial cells. The amino acid sequences of topoisomerase I in mammalian, fungal and bacterial cells were shown in Additional file 1: Table S1, Additional file 2: Fig. S1. Interestingly, the dual activity of camptothecin as anticancer and antimicrobial is one of the most fascinating biological criteria in cancer therapy, since the chemotherapy “camptothecin medication” cause suppression to the human immune system, allowing to the opportunistic microbial flora to be a pathogenic and virulent microbes [43]. This hypothesis has been confirmed from the explored shared properties of tumor and fungal cells such as high replication rates, virulence, modalities of spreading within the host, rapid development of drug-resistance mechanisms, and tendency to become more aggressive during disease progression [43]. Thus, the higher dual antiproliferative and antifungal activity of extracted *A. terreus* camptothecin seems to be a superior biological feature for the clinical applications.

### Bioprocess optimization of camptothecin production by *A. terreus* with Plackett–Burman design

**Fig. 5:**
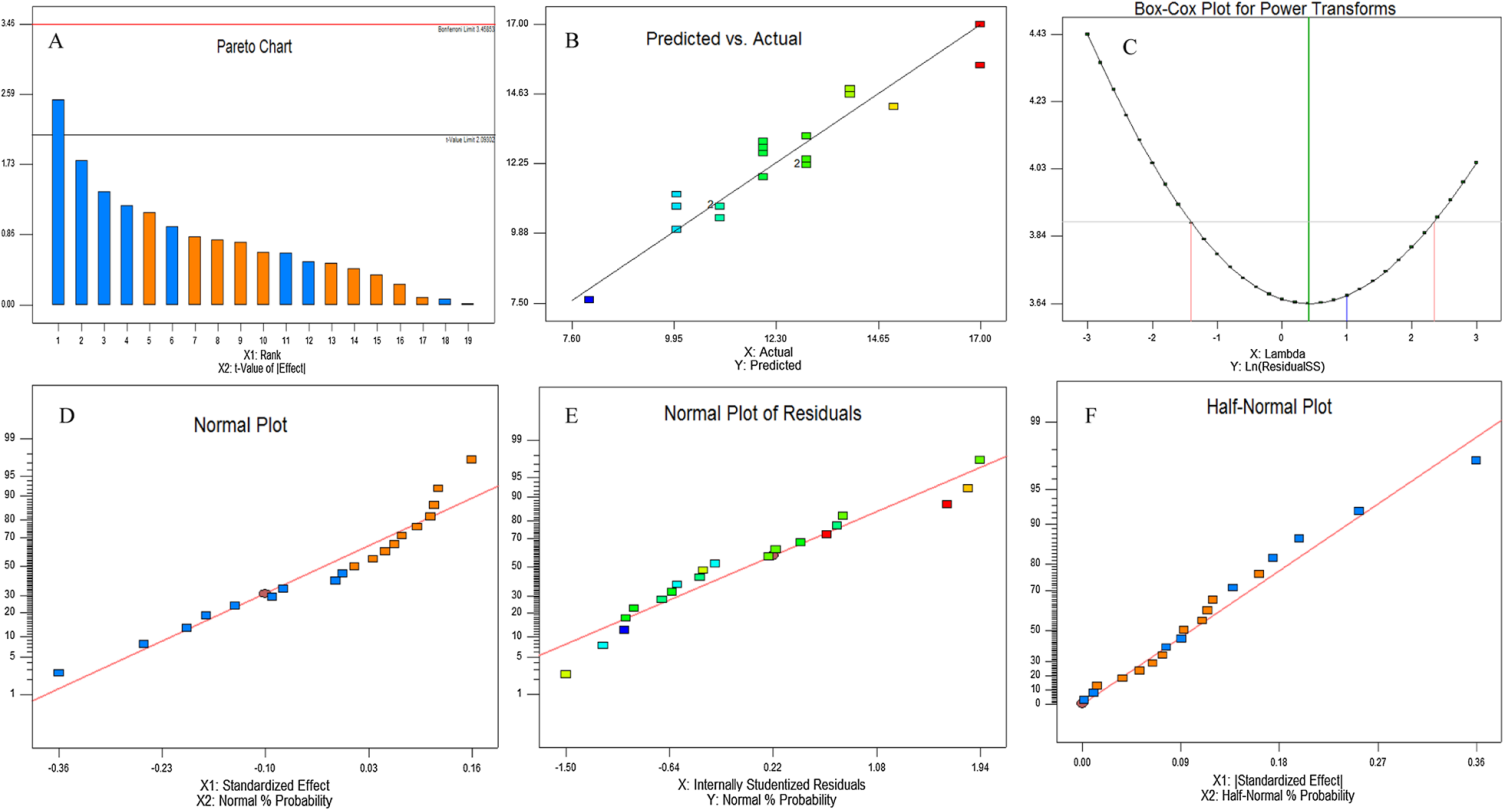
Bioprocess optimization of camptothecin production by *A. terreus* with the Plackett–Burman experimental design. **A** Pareto chart illustrating the significance of each variable. **B** Plot of correlation of the predicted and actual camptothecin yield of *A. terreus.* **C** Box-Cox power transform. Plot of standardized effect (**D**), internal standardized residuals (**E**) with the normal probability. **F** Plot of standardized effect with the half-normal probability

**Fig. 6:**
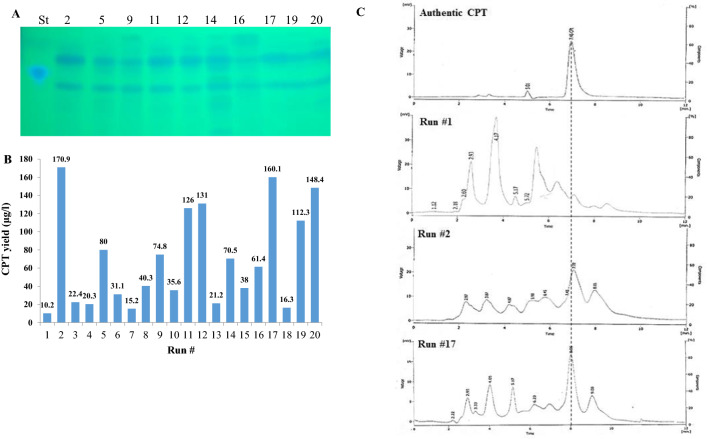
The yield of camptothecin by *A. terreus* in response to optimization bioprocessing by Plackett–Burman design. The fungal cultures grown on the corresponding medium components, incubated, and the camptothecin were extracted, and quantified. The TLC chromatogram (**A**) and yield of the extracted camptothecin (**B**), as determined by the Image J software package. C, HPLC chromatogram of the most potent runs for camptothecin production (Run #2, Run #3) comparing to lowest ones (Run #1), normalizing to authentic camptothecin

*Aspergillus terreus* has been selected for further nutritional optimization to maximize their yield of camptothecin, since the identity of medium chemical components and their interactions are pivotal for synthesis of secondary metabolites by fungi. The nutritional requirements for camptothecin production by *A. terreus* were optimized by Plackett–Burman design as “1st order model equation” to determine the significant factors affecting camptothecin productivity. Nineteen variables including the different carbon, and nitrogen sources as precursors of camptothecin, in addition to the growth modulators and elicitors of *A. terreus* were studied, with their lower (− 1) and higher (+ 1) values regarding to camptothecin productivity (Table 1). The significance of the tested independent variables influencing camptothecin productivity by *A. terreus*, with the predicted and corresponding actual responses, was summarized in the Plackett–Burman design matrix (Table 2). The value of the coefficient of determination (R2 = 0.98) indicating a goodness-of-fit measure for the linear regression models. The analysis of variance (ANOVA) of the experimental design was calculated, and the coefficients, t Stat, *p*-value, and confidence levels were recorded (Table 3). The variability in the response (98.87%) was attributed to the selected independent variables. Additionally, the F-value (9.87) and *p*-value (< 0.0007), and adjusted determination coefficient (Adj. R2 = 0.92) indicated the significance of this model. The main effects and the normal probability of the examined factors were measured and plotted, indicating that there are four different independent factors; potato extract, salicylic acid, cysteine, and glutamate that have a positive effect on the yield of camptothecin, while the other independent factors have no effect. The highest yield of CPT (170.09 μg/L) was recorded in run 2, while the lowest value (10.5 μg/L) was measured in run 7. The significance of the variables affecting camptothecin productivity by *A. terreus* has been displayed in the Pareto Chart, as well as the probability plot of independent variables, actual and predicted yield of camptothecin (Fig. 5). The arrangement of the points of residuals around the diagonal line shows the independent normal distribution of the variables, suggesting the perfect fitting of predicted and actual camptothecin yield. From the ANOVA analysis, the constructed model was highly significant, as shown from the values of Fisher’s F-test 9.87 and probability p-value 0.0007. The yield of camptothecin by *A. terreus* for the highest experimental runs #2, 11, 12 was quantified by HPLC, normalizing to authentic camptothecin (Fig. 6). The actual and predicted yield of camptothecin by *A. terreus* was noticeably fluctuated from 10.5 to 170.5 μg/L, confirming the significance of the tested variables on camptothecin biosynthesis, and the efficiency of the Plackett–Burman design, the values of the coefficient of determination (R2 = 0.98) indicating the goodness-of-fit measure for the linear regression models (Table 3). The first order polynomial equation of camptothecin production by *A. terreus,* regarding the significant independent variables was derived from the following equation:

$$\begin{aligned}& {\text{Yield of camptothecin by }}A. \, terreus\, \\&= \,{22}.0{6667}\, + \,0.{\text{16*Acid whey}} \\ &\quad- 0.{46667}*{\text{Diaion HP2}}0\, \\ & \quad+ \,0.{\text{26667*Amberlite XAD}}\\ &\quad - 0.{36}*{\text{dextrin}}\, + \,0.{12}*{\text{glucose }} \\ &\quad - {1}.{3}*{\text{ salicylic acid}}\, + \,0.{2}*{\text{serine}}\\ & \quad- 0.{16667}*{\text{cysteine}} - 0.{12}*{\text{glutamate}}{.} \\ \end{aligned}$$

**Table 1:** The coded and actual values for the tested variables affecting CPT production by *Aspergillus terreus*

Codes	Factors	Levels	
		− 1	1
X1	Acid whey (mL/L)	2	5
X2	Malt extract (g/L)	2	5
X3	Potato starch (g/L)	10	20
X4	Diaion HP20 (g/L)	2	5
X5	Amberlite XAD (g/L)	2	5
X6	Methyljasmonate (g/L)	0.1	0.5
X7	Tryptamine (g/L)	2	4
X8	Peptone (g/L)	5	10
X9	Dextrin (g/L)	5	10
X10	Tryptone (g/L)	10	20
X11	Glucose (g/L)	10	20
X12	Salicylic Acid (g/L)	2	4
X13	Tryptophan (g/L)	4	10
X14	Serine (g/L)	4	8
X15	Cysteine (g/L)	4	10
X16	Pyruvate (g/L)	5	10
X17	Phenylalanine (g/L)	4	8
X18	Glutamate (g/L)	10	20
X19	Fluconazole (g/L)	2	5

**Table 2:** Plackett–Burman experimental design for evaluating the effect of nineteen-independent variables on the actual and predicted camptothecin yield from *Aspergillus terreus*

Run	X1	X2	X3	X4	X5	X6	X7	X8	X9	X10	X11	X12	X13	X14	X15	X16	X17	X18	X19	Actual yield	Predicted yield	Residuals
1	10	2	20	2	2	0.1	2	10	20	10	20	4	4	4	10	10	8	20	2	50.02	48.50	− 1.486
2	5	5	10	2	2	0.1	4	10	15	20	20	2	4	8	10	10	8	10	5	170.09	168.33	1.727
3	5	5	20	2	5	0.5	2	5	20	20	20	4	4	8	4	10	4	10	2	20.24	20.50	− 0.266
4	5	2	20	5	2	0.5	4	5	15	20	20	4	10	4	10	5	8	10	2	20.03	20.50	− 0.476
5	5	5	20	5	5	0.1	4	5	20	10	10	2	4	8	10	5	8	20	2	80	110.36	− 3.363
6	10	5	10	5	2	0.5	2	5	15	10	20	4	4	8	10	5	4	20	5	30.11	20.50	0.604
7	10	5	20	2	5	0.1	4	5	15	10	10	4	10	4	10	10	4	10	5	10.52	20.50	− 0.986
8	10	5	10	2	5	0.5	4	10	15	20	10	4	4	4	4	5	8	20	2	40.03	20.50	1.524
9	5	2	20	5	5	0.5	2	10	15	20	10	2	4	4	10	10	4	20	5	70.48	110.36	− 3.883
10	5	5	10	5	2	0.1	2	5	20	20	10	4	10	4	4	10	8	20	5	30.56	20.50	1.054
11	10	5	10	5	5	0.1	2	10	20	20	20	2	10	4	10	5	4	10	2	120.6	110.36	1.237
12	10	5	20	5	2	0.5	2	10	15	10	10	2	10	8	4	10	8	10	2	130.1	110.36	1.827
13	5	2	10	2	5	0.5	2	10	20	10	10	4	10	8	10	5	8	10	5	20.12	20.50	− 0.386
14	5	5	20	2	2	0.5	4	10	20	10	20	2	10	4	4	5	4	20	5	70.05	110.36	− 4.313
15	10	2	20	5	2	0.1	4	10	20	20	10	4	4	8	4	5	4	10	5	30.8	20.50	1.294
16	10	2	10	2	2	0.5	4	5	20	20	10	2	10	8	10	10	4	20	2	60.14	110.36	− 5.223
17	5	2	10	2	2	0.1	2	5	15	10	10	2	4	4	4	5	4	10	2	160.01	110.36	4.647
18	5	2	10	5	5	0.1	4	10	15	10	20	4	10	8	4	10	4	20	2	10.63	20.50	− 0.876
19	10	2	20	2	5	0.1	2	5	15	20	20	2	10	8	4	5	8	20	5	110.23	110.36	− 0.133
20	10	2	10	5	5	0.5	4	5	20	10	20	2	4	4	4	10	8	10	5	140.84	110.96	3.477

The “−1′′ sign correspond to the minimum value and the “+1′′ sign correspond to the maximum value of the input parameter range

**Table 3:** Regression statistics and analysis of variance (ANOVA) for Placket-Burman design

Source	Sum of squares	df	Mean square	F-value	p-value Prob > F	
Model	88.8	9	9.87	9.87	0.0007	Significant
A-Acid Whey	3.2	1	3.2	3.2	0.1039	
D-Diaion HP20	9.8	1	9.8	9.8	0.0107	
E-Amberlite XAD	3.2	1	3.2	3.2	0.1039	
J-Dextrin	16.2	1	16.2	16.2	0.0024	
L-Glucose	7.2	1	7.2	7.2	0.023	
M-Salicylic acid	33.8	1	33.8	33.8	0.0002	
O-Serine	3.2	1	3.2	3.2	0.1039	
P-Cysteine	5	1	5	5	0.0493	
S-Glutamate	7.2	1	7.2	7.2	0.023	
Residual	10	10	1			
Cor Total	98.8	19				

The Model F-value of 28.86 implies the model is significant. There is only a 0.01% chance that a "Model F-Value" this large could occur due to noise

Values of "Prob > F" less than 0.0500 indicate model terms are significant

In this case C, M, S are significant model terms

Values greater than 0.1000 indicate the model terms are not significant

If there are many insignificant model terms (not counting those required to support hierarchy), model reduction may improve your model

The highest correlation between the predicted and observed values indicated the validity of the statistical design. By using the regression model, the highest actual yield of camptothecin yield by *A. terreus* was 170.1 μg/L, that close to the predicted one 186.3 μg/L which proved the validity of this model (Fig. 6). Based on the Plackett–Burman Design, the optimal medium components for maximum yield of camptothecin by *A. terreus* contains acid why (2 mL/L), Diaion HP20 (2 g/L), amberlite XAD (2 g/L), dextrin (5 g/L), glucose (10 g/L), salicylic acid (2 g/L), serine (4 g/L), cysteine (4 g/L) and glutamate (10 g/L), at pH 6, after 15 days of incubation. Thus, based on this optimized medium, the actual yield of camptothecin by *A. terreus* (170.1 μg/L) was increased by 1.6 folds than the basal medium (110 μg/L). Similar results were obtained for camptothecin nutritional optimization bioprocessing with Plackett–Burman Design by *A. flavus*, *A. terreus* and *Penicillium chrysogenum* [10, 34, 35].

### Biosynthetic stability of camptothecin by subculturing of *A. terreus*

**Fig. 7:**
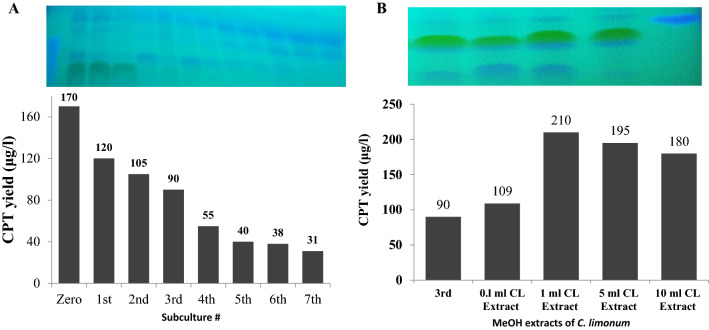
Metabolic stability of *A. terreus* for sustainable production of camptothecin with the multiple subculturing. **A** The yield of camptothecin with the subsequent cultural generations *of A. terreus*. **B** The 3rd generation of *A. terreus* was cultured on the optimized medium, amended with different concentrations of lemon peel extracts (0.5–10 mL), incubated at standard conditions, then camptothecin was extracted and quantified

The biosynthetic stability of camptothecin productivity by *A. terreus* in response to multiple subculturing was evaluated. The axenic first fungal isolate, was maintained as slope culture on PDA for 8 days at 30 °C, then subsequently sub-cultured under the same conditions till the 7th generation, and their camptothecin productivity was determined. From the results (Fig. 7), the camptothecin productivity by *A. terreus* was decreased with successive subculturing of the fungus. The yield of camptothecin of the zero culture of *A. terreus* (170.1 µg/L) was suppressed by ~ 3 times by the 4th generation (55.1 µg/L), and by ~ 5.7 folds by the 7th generation (31 µg/L). This metabolic attenuation of camptothecin productivity by *A. terreus* has been reported as a common physiological criterion for various camptothecin producing endophytic fungi and endozoic fungi [16, 26, 27, 42]. Obviously, the relative biosynthetic stability of camptothecin by the endophytic fungus could be due to the stability of the associated inducing signals derived from the plant host with the fungal spores, and these signals might be diluted with the successive generations [8, 14]. The attenuation of camptothecin biosynthesis could be attributed to the dilution of the chemical signals triggering the expression of camptothecin encoding gene cluster from the host plants, lack of cultural communication and cross-talking among the camptothecin producing and non-producing microbial endophytes [8, 14].

### Restoring the camptothecin biosynthetic machinery of *A. terreus* by addition of lemon peels extracts

Interestingly, in an endeavor to the restore the biosynthetic stability of camptothecin by *A. terreus,* the 3rd cultural generation of *A. terreus* was amended with different concentrations of methanolic extract of *C. limonum* peels. After incubation, the cultures were filtered and camptothecin productivity was checked by TLC and HPLC. From the results (Fig. 7B), a dramatic activation of the biosynthetic machinery of camptothecin by *A. terreus* was observed at 1 mL of methanolic lemon peel extract/ 50 mL medium, has been observed, by about 2.2 increment folds (210 µg/L), regarding to the 3rd fungal generation (90 µg/L). Thus, upon addition of extract of lemon peel, the biosynthetic machinery of camptothecin has been completely restored, ensuring the presence of some chemical signals or compounds that could be implemented by the fungus as intermediates on the biosynthetic pathways of camptothecin. In addition, the stimulatory effect of lemon peel extract could be due to the presence of some epigenetic regulators, modifiers that modulate the expression of the camptothecin-biosynthetic gene clusters, as evidenced for Taxol production by fungal-bacterial cocultivation [10, 11]. The major components of lemon peel extract are limonene was found to be the major constituent (43%), β-pinene (12%), gamma terpinene (11%), α-terpineol (7%), α-pinene (3%), myrecene (1%), geraniol (1%), α-terpinene (1.32%), α-terpinolene (2.37%), linalool (1.08%) and cis-α-bergamotene(1.38%) [44]. Lemon plant extract exhibited a broad spectrum biological activities such as, anti-inflammatory, antimicrobial anticancer antioxidant activity [44, 45]. Citral is the main component of peel lemon extract, a mixture of neral and geranial monoterpene aldehydes [46], that exhibited a potent antimicrobial activity [47]. The compounds with antimicrobial activity of the lemon peel extract, might be the signals or transcriptional factors triggering the expression the camptothecin encoding orphan-genes, further proteomic and metabolomics analysis are ongoing to elucidate the biological and chemical identity of these signals and the identity of the overexpressed genes related to camptothecin biosynthetic machinery.

In conclusion, *Aspergillus terreus* ON908494.1, an endophyte of *Cestrum parqui,* was identified with relative metabolic sustainability for camptothecin production (170 µg/L). The chemical identity of *A. terreus* camptothecin was resolved from the HPLC, FTIR and LC–MS/MS analyses. The putative camptothecin displayed a strong dual bioactivity as anticancer agent against HepG-2 and MCF-7, and antibacterial and antifungal activity. The productivity of *A. terreus* camptothecin was slightly reduced with the fungal subculturing, however, the fungal biosynthetic potency of camptothecin has been completely restored upon addition of lemon peel extracts. The isolated strain can be used to develop a microbial-based sustainable bioprocess for large scale production of camptothecin for cancer therapy.

## Supplementary Information

**Additional file 1:** **Table S1. **Screening for Camptothecin production from the endophytic fungi inhabiting different *Cestrum* species.

**Additional file 2: Figure S1.** Phylogenetic relatedness of the amino acid sequences of the Topoisomerase I from different microorganisms and Homo sapiens by MEGA-X Software package.

## Data Availability

All datasets generated for this study are included in the manuscript.
