# Supplementary material for: Production and bioprocessing of camptothecin from Aspergillus terreus, an endophyte of Cestrum parqui, restoring their biosynthetic potency by Citrus limonum peel extracts
Source: Microb Cell Fact. 2023 Jan 6;22:4. doi: 10.1186/s12934-022-02012-y (PMC9824926; doi:10.1186/s12934-022-02012-y)
Supplement: Supplementary file 1 — Additional file 1: Table S1. Screening for Camptothecin production from the endophytic fungi inhabiting different Cestrum species. [file 12934_2022_2012_MOESM1_ESM.docx]

**Table S1. Screening for Camptothecin production from the endophytic fungi inhabiting different *Cestrum* species**

| Camptothecin Yield  ( µg) | Camptothecin spot intensity on TLC | Fungal isolate | Isolate # | Plant part | Host Plant |
| --- | --- | --- | --- | --- | --- |
| **60** | **+** | Aspergillus niger | 1 | Leaf | *Cestrum diurnum* |
| **80** | **++** | Aspergillus flavus links | 2 |  |  |
| **20** | **-** | Aspergillus fumigatus | 3 |  |  |
| **40** | **+** | Aspergillus niger | 4 | Stem |  |
| **89** | **++** | Aspergillus flavus links | 5 |  |  |
| **0** | **-** | Aspergillus oryzae | 6 |  |  |
| **40** | **+** | Aspergillus fumigatus | 7 |  |  |
| **0** | **-** | Aspergillus niger | 8 | Leaf | *Cestrum elegans* |
| **56** | **++** | Aspergillus flavus links | 9 |  |  |
| **80** | **++** | Aspergillus flavus columnaris | 10 |  |  |
| **0** | **-** | Aspergillus niger | 11 | Stem |  |
| **0** | **-** | Aspergillus awamorii | 12 |  |  |
| **40** | **+** | Aspergillus flavus links | 13 |  |  |
| **35** | **+** | Aspergillus niger | 14 | Leaf | *Cestrum nocturnum* |
| **0** | **-** | Aspergillus flavus links | 15 |  |  |
| **39** | **+** | Aspergillus niger | 16 | Stem |  |
| **34** | **+** | Aspergillus oryzae | 17 |  |  |
| **0** | **-** | Aspergillus parasiticus | 18 |  |  |
| **38** | **++** | Aspergillus fumigatus | 19 |  |  |
| **98** | **+++** | Aspergillus terreus | 20 |  |  |
| **70** | **+** | Aspergillus niger | 21 | Leaf | *Cestrum parqui* |
| **62** | **+** | Aspergillus flavus links | 22 |  |  |
| **0** | **-** | Aspergillus flavus columnaris | 23 |  |  |
| **96** | **+++** | Aspergillus terreus | 24 |  |  |
| **0** | **-** | Aspergillus ustus | 25 |  |  |
| **22** | **+** | Aspergillus fumigatus | 26 |  |  |
| **110** | **++++** | Aspergillus terreus | 27 |  |  |
| **0** | **-** | Aspergillus niger | 28 | Stem |  |
| **0** | **-** | Aspergillus awamorii | 29 |  |  |
| **0** | **-** | Aspergillus flavus links | 30 |  |  |
| **0** | **-** | Aspergillus fumigatus | 31 |  |  |
