# Supplementary figures and images for: Production and bioprocessing of camptothecin from Aspergillus terreus, an endophyte of Cestrum parqui, restoring their biosynthetic potency by Citrus limonum peel extracts
Source: Microb Cell Fact. 2023 Jan 6;22:4. doi: 10.1186/s12934-022-02012-y (PMC9824926; doi:10.1186/s12934-022-02012-y)

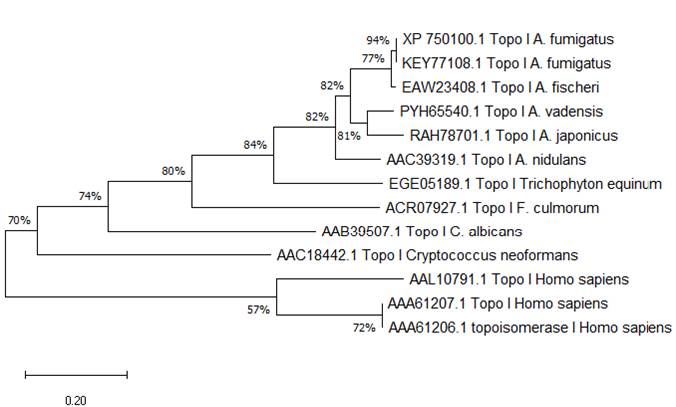

Supplement: Supplementary file 2 — Additional file 2: Figure S1. Phylogenetic relatedness of the amino acid sequences of the Topoisomerase I from different microorganisms and Homo sapiens by MEGA-X Software package. [file 12934_2022_2012_MOESM2_ESM.jpg]
